# Household

**Published:** 1886-12

**Authors:** 


### HOUSEHOLD.

TO REMOVE OLD INK SPOTS.—Pyrophosphate of soda is recommended, when the stains are on colored fabrics, as the salt does not injure their color.

LOTIONS FOR FRECKLES.—The following are both good and harmless cosmetics:

(1) Borax, 3 grs.
Rose water, 5 dr.
Orange flower-water, 5 dr.
(2) Orange flower-water, 1 pint.
Glycerine, 1 oz.
Borax, 1 dr.

MOTH PREVENTIVE.—The following recipe for keeping moths out of clothing is a favorite in some families: Mix half a pint of alcohol, the same quantity of spirits of turpentine, and two ounces of camphor. Keep in a stone bottle, and shake before using. The clothes or furs are to be wrapped in linen, and crumpled-up pieces of blotting paper dipped in the liquid are to be placed in the box with them, so that it smells strong. This requires renewing about once a year.

---

HOUSEHOLD HINTS.—If the oven is too hot when baking, place a small dish of cold water in it.

When sponge cake becomes dry it is nice to cut in thin slices and toast.

Whitewashed walls can be papered by first washing with vinegar to "kill" the lime.

Iron rust can be removed from clothes by rubbing with lemon juice and laying in the sun.

Sunshine on mirrors will injure their lustre, therefore do not hang opposite a door or window.

Hot alum is the best insect destroyer known. Put it in hot water, and let it boil until all the alum is dissolved. Apply hot, with a brush, and all creeping things are instantly destroyed.

---

FACTS WORTH KNOWING.—Red ants will never be found in closets or drawers if a small bag of sulphur is kept in these places.

To harden cast iron, mix one-half pint of oil of vitriol and two ounces of saltpetre in three gallons of clean water. Heat the iron to a cherry red, and dip as usual.

When larger flower pots are used, there will be more leaves than flowers. Often plants do not bloom because, having so much space, their strength is expended in forming roots and leaves.

It has been discovered by a Chicago physician that suburban life is a powerful provocative of dyspepsia. Men are like animals, and must eat their meals quietly and leisurely to secure a perfect flow of the gastric juice.

It is said that watercress destroys the toxic principle of tobacco without destroying its other qualities. If this information can be relied on, smokers have only to moisten their tobacco with the juice of watercresses and they can enjoy a harmless smoke.

---

TO DEVELOP THE LUNGS.—If a person's lungs are not well developed, the health will be imperfect, but the development may be increased several inches in a few months by daily out-door runnings with the mouth closed, beginning with twenty yards and back, at a time, increasing ten yards every week, until a hundred are gone over thrice a day. A substitute for ladies and persons in cities, is running up stairs with the mouth closed, which compels very deep inspirations, in a natural way, at the end of the journey.

---

To Preserve Paste and Glue.—It is found that the unwholesome and offensive effluvia from decomposing paste and glue can be overcome by a very simple method. If, when making the paste and glue, a small quantity of carbolic acid is added to it, it will continue in a sweet condition to the end. A few drops added to mucilage or ink prevents mould. Again, in whitewashing the cellar, dairy, etc., if an ounce of carbolic acid is added to each gallon of wash, it will prevent mould, and also the disagreeable taints often perceived in milk and meats from damp apartments. Another peculiar advantage claimed for the use of carbolic acid in paste for wall paper and whitewash is that it will drive away insect pests. The cheapest and best form of carbolic acid is the crystals, which dissolve in water, or liquify at an excess of temperature.

The following is the goverment method of cleaning brass, in use at all the United States arsenals, which is claimed to be the best in the world: The plan is to make a mixture of one part common nitric acid and one-half sulphuric acid in a stone jar, having also ready a pail of fresh water and a box of sawdust. The articles to be treated are dipped into the acid, then removed into the water, and finally rubbed with the sawdust. This immediately changes them to a brilliant color. If the brass has become greasy, it is first dipped in a strong solution of potash and soda in warm water; this cuts the grease so that the acid has free power to act.

---

Stoved potatoes is a dish which might be introduced on the tables of vegetarians with advantage; it is one of the nicest ways of cooking the potato, any kind of which may be used, though personally I prefer them cooked in this way during June and July, when they have outgrown the new waxy stage, but are not yet old. Three-quarters of a panful are peeled or scraped and cut into slices about a quarter of an inch thick; a bunch of green onions cut in short lengths is added, a little salt, and plenty of pepper; and, lastly, a large breakfast-cupful of sweet rich cream is poured in, the saucepan lid put on tightly, and the pan placed over a slow fire. It is stirred occasionally to prevent burning, but the lid is always put tightly on again. It will cook in about half an hour. This is served either alone or with a chop or steak. Another way is to use chopped suet instead of cream, when cream cannot be procured, or economy is an object.

---

Vinegar and Indigestion.—It is the business of the saliva to digest starch, and by its alkalinity, to stimulate the secretion of the gastric juice in the stomach. It is well known that the saliva is unable to act upon starch in the presence of an acid. Experiments have shown that even so small a quantity of vinegar as one part in 5,000 appreciably diminishes the action of saliva upon starch, One part in 1,000 renders it very slow, and twice the latter quantity arrests it altogether. From this it is evident that vinegar, pickles, salads, and other preparations in which vinegar is used are unwholesome, especially when taken with farinaceous food, such as bread and other grain preparations. There is a popular notion that by the use of vinegar a tendency to increase in flesh may be antagonized. The physiological fact that fat is largely formed from the starchy elements of grains and vegetables, rather supports the popular notion; but this method of reducing weight should not be encouraged, as the loss of flesh is secured at the expense of good digestion.—*Good Health.*

